# Supplementary material for: Adsorption Equilibrium and Mechanism and of Water Molecule on the Surfaces of Molybdenite (MoS2) Based on Kinetic Monte-Carlo Method
Source: Molecules. 2022 Dec 8;27(24):8710. doi: 10.3390/molecules27248710 (PMC9785587; doi:10.3390/molecules27248710)
Supplement: Supplementary file 1 [file molecules-27-08710-s001.zip › molecules-1901708-supplementary.pdf]

Section S1. Desorption rate of H_2 from MoS_2 unit cell based on geometry optimization

According to the lattice constant and band-gap calculation, we choose the PBE functional and cut off energy of 381 ev, the K-point as 5×5×1 to accomplish the geometry optimization of the reactant (H adsorbed on MoS_2) and product (H desorbed from MoS_2) to obtain the background state and calculate the enthalpy in the vacancy at 0 K. Equation S1 can be used to relate the energy change and the equilibrium constant.

$$\Delta G_r^0 = \Delta H_r^0 - T\Delta S_r^0 = -RT \ln K^0 \qquad \text{(Equation S1)}$$

$$\ln K^0 \approx \frac{(\Delta H^0_{Product} - \Delta H^0_{Reactant})}{-RT} \qquad \text{(Equation S2)}$$

In the small slab, the entropy contribution was assumed as zero ($S^0_r \approx 0$). Worth to be noted that the energy change of this reaction corresponds to the reaction S1, which should multiply the coefficient to estimate the equilibrium constant of the reaction S2:

H-MoS_2 = MoS_2 + H (Reaction S1)

2H-MoS_2 = MoS_2 + 2H (Reaction S2)

Total enthalpy of reactant: −1.57011002E+003 eV

Total enthalpy of product: −1.57010972E+003 eV

According to the equation (4) and A-site derived from sorption isotherm, we can obtain that: $K_{H2\text{-}adsorption} = 1.20 \times 10^8\ s^{-1}$

The equilibrium constant K_{eq} of reaction S1 at 298 K equals to 0.99. According to the equation 5, we can obtain that: $K_{H2\text{-}desorption} = 2.38 \times 10^8\ s^{-1}$

Section S2. Formation and dissociation rate coefficient of H_2O above MoS_2

The dissociation of H_2O above MoS_2 can be expressed in an elementary reaction S3.

MoS_2-H_2O = MoS_2-OH + H (Reaction S3)

$\Delta G^0_{(reaction\text{-}S3)}$=0.06 eV (Ghuman et al., 2015) ; K_{eq} of Reaction S3 ($K_{eq\text{-}reaction\text{-}S3}$) equals to 0.096768361.

$K_{eq\text{-}reaction\text{-}S3}$ = KH_2O-dissocation/KH_2O-formation

$K_{H2O\text{-}dissociation} = 2.8 \times 10^{10}\ S^{-1}$ (Ghuman et al., 2015)

According to the equation (5), we can obtain that:

$K_{H2O\text{-}formation} = 2.9 \times 10^{11}\ S^{-1}$

Section S3. Adsorption and desorption rate coefficient of H_2O above MoS_2

The adsorption of H_2O above MoS_2 can be expressed in an elementary reaction S4.

MoS_2 + H_2O = MoS_2-H_2O (Reaction S4)

$\Delta G^0_{(reaction\text{-}S4)}$ = 0.17 eV (Ghuman et al., 2015) ; K_{eq} of reaction-S4 ($K_{eq\text{-}reaction\text{-}S4}$) equals to 0.001337348

$K_{eq\text{-}reaction\text{-}S4}$ = KH_2O-adsorption/KH_2O-desorption

$K_{H2O\text{-}adsorption} = 4.84 \times 10^7\ S^{-1}$ (Ghuman et al., 2015)

According to the equation (5), we can obtain that:

$K_{H2O\text{-}desorption} = 3.62 \times 10^{10}\ S^{-1}$
